# Maternal pre-pregnancy body mass index, smoking in pregnancy, and alcohol intake in pregnancy in relation to pubertal timing in the children

**DOI:** 10.1186/s12887-019-1715-0

**Published:** 2019-09-16

**Authors:** Nis Brix, Andreas Ernst, Lea Lykke Braskhøj Lauridsen, Erik Thorlund Parner, Onyebuchi A. Arah, Jørn Olsen, Tine Brink Henriksen, Cecilia Høst Ramlau-Hansen

**Affiliations:** 1 Department of Public Health, Section for Epidemiology, Aarhus University, Bartholins Allé 2, 8000 Aarhus C, Denmark; 2 Department of Epidemiology, Fielding School of Public Health, University of California Los Angeles (UCLA), Los Angeles, CA 90095-1772 USA; 3 Department of Public Health, Section for Biostatistics, Aarhus University, DK-8000 Aarhus, Denmark; 4 Department of Statistics, UCLA College of Letters and Science, Los Angeles, CA 90095-1554 USA; 5 Department of Clinical Epidemiology, Aarhus University Hospital, DK-8200 Aarhus, Denmark; 6 Perinatal Epidemiology Research Unit, Department of Paediatrics, Aarhus University Hospital, DK-8200 Aarhus, Denmark

**Keywords:** Puberty, Marker of pubertal timing, Prenatal exposures, Smoking, Alcohol consumption, Obesity, Age at menarche

## Abstract

**Background:**

Earlier pubertal timing has been observed in many countries. We aimed to explore if prenatal exposure to maternal obesity, smoking, and alcohol intake was associated with timing of puberty by use of a novel marker of pubertal timing: ‘the height difference in standard deviations’ (HD:SDS).

**Methods:**

HD:SDS is the difference between pubertal height in standard deviations and adult height in standard deviations, and it correlates well with age at peak height velocity. Pubertal height was measured by health care professionals at approximately 13 years in boys and 11 years in girls, and the children’s adult height was predicted from parental height reported by the mothers during pregnancy. Information on HD:SDS was available for 42,849 of 56,641 eligible boys and girls from the Danish National Birth Cohort born 2000–2003. In a subsample, HD:SDS was validated against age at the following self-reported pubertal milestones: Tanner stages, menarche, first ejaculation, voice break, acne, and axillary hair. Prenatal exposures were reported by mothers during pregnancy.

**Results:**

HD:SDS correlated moderately with the pubertal milestones considered (correlation coefficients: − 0.20 to − 0.53). With normal weight (body mass index (BMI): 18.5–24.9 kg/m^2^) as the reference, maternal pre-pregnancy obesity (BMI: 30.0+ kg/m^2^) was associated with earlier pubertal timing: 0.23 (95% confidence interval (CI): 0.18, 0.28) higher HD:SDS in boys and 0.19 (95% CI, 0.14, 0.24) higher HD:SDS in girls. Maternal smoking was not associated with pubertal timing. Compared to alcohol abstainers, maternal intake of > 3 units of alcohol weekly was associated with later puberty in boys only: 0.14 (95% CI, 0.05, 0.24) lower HD:SDS.

**Conclusion:**

As correlations between HD:SDS and the considered pubertal milestones were comparable to those reported in the literature between age a peak height velocity and the considered pubertal milestones, the validity of HD:SDS seems acceptable. Maternal pre-pregnancy obesity was associated with earlier pubertal timing in both sexes, and maternal alcohol intake during pregnancy was associated with later pubertal timing in boys. Maternal smoking has been linked to earlier timing of puberty, but this was not replicated in our setting using HD:SDS as a marker of pubertal timing.

## Background

During the last century, a trend toward earlier age at onset of puberty has been reported in girls, whereas the trend toward earlier age at menarche has leveled off in some countries since the 1960s [1, 2]. A trend is less clear in boys [2]. A change in timing of puberty over time is of concern because early pubertal timing has been associated with increased risk of adult diseases such as type 2 diabetes, cardiovascular diseases, testicular cancer, and breast cancer [3]. Identification of modifiable risk factors for earlier pubertal timing is, therefore, warranted. A potential modifiable risk factor for earlier pubertal timing is childhood obesity, which has been suggested to be responsible for some of the trend [4, 5]. Prenatal exposures have also been suggested to interfere with pubertal timing through fetal growth restriction or overnutrition as well as endocrine disruption of androgenic or estrogenic activity [5, 6]. Maternal obesity, smoking, and alcohol intake during pregnancy may lead to either fetal growth restriction [7, 8] or overnutrition [9] and may change the intrauterine hormonal milieu towards either increased androgenic activity [10] or increased estrogenic activity [11, 12]. Hence, these maternal exposures may potentially interfere with pubertal timing in the children. In girls, earlier puberty has been observed after prenatal exposure to maternal obesity [13–19] and smoking [20–23]. No association for prenatal exposure to alcohol has been reported in girls in all [22, 24–26] but one small study [27]. In boys, some evidence support an earlier pubertal timing after prenatal exposure to smoking [23, 28–30], whereas results has been inconclusive for prenatal exposure to maternal obesity [31] and prenatal alcohol consumption [26, 32, 33]. If these associations reflect causal relations, they may open up for a potential for preventive actions as they are relatively frequent and modifiable [34–36].

Several markers of pubertal timing exist, but they all have limitations [2]: self-reported Tanner stages may be prone to misclassification, clinical assessment of Tanner stages may have high non-participation, age at peak height velocity (PHV) requires repeated height measures on each child, and hormonal analyses are expensive. Wehkalampi et al. developed a novel, low-cost, and simple marker of pubertal timing, namely ‘the height difference in standard deviations’ (HD:SDS) [37]. HD:SDS is the difference between pubertal height (measured around the mean age at PHV on the population level) in standard deviation scores (SDS) and adult height in SDS [37]. The rationale is that pubertal height is assumed to be mainly determined by the pubertal timing and the genetic growth potential, whereas adult height is assumed to be mainly determined by the genetic growth potential. By subtracting adult height SDS from pubertal height SDS, the genetic contribution of pubertal height is considered removed. The resulting HD:SDS is, in theory, only influenced by pubertal timing [37]. This is corroborated by empirical data revealing high correlation between HD:SDS and age at PHV [37].

We validated a modified version of HD:SDS. The modified HD:SDS relied on adult height predicted from parental height reported by the mothers during pregnancy because the children were not fully grown yet. Then, we explored how prenatal exposure to maternal obesity, smoking, and alcohol consumption were associated with timing of puberty, measured by HD:SDS, in boys and girls.

## Methods

### Study population

**Fig. 1:**
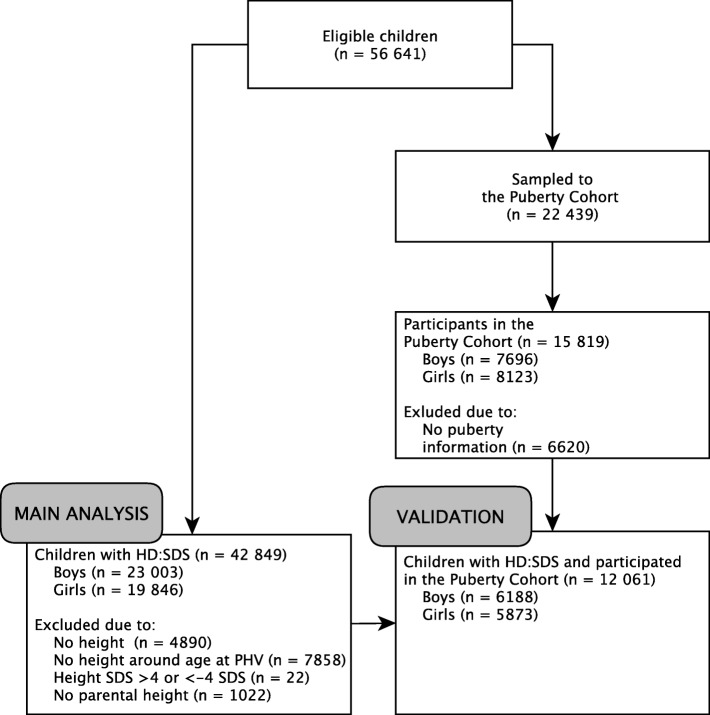
Flow diagram of children in the main analysis and in the validation analysis of HD:SDS, the Danish National Birth Cohort, Denmark. Abbreviations: HD:SDS, height difference in standard deviations; PHV, peak height velocity; SDS, standard deviation score

This population-based cohort study was based on the Danish National Birth Cohort (DNBC) [38]. The DNBC contains approximately 100,000 children and their mothers, who were recruited during early gestation in 1996–2002. Mothers were interviewed twice during pregnancy around week 17 and 32 of gestation, and their children were followed up at 0.5, 1.5, 7 and 11 years of age. Mother-child pairs were eligible for the present study if the children were singletons born during 2000–2003, whose mothers had participated in the first computer-assisted telephone interview shortly after recruitment and had not withdrawn from the DNBC (*n* = 56,641) (Fig. 1).

### Height difference in standard deviations (HD:SDS)

The height SDS describes how far (in standard deviations) a person’s actual height is from that person’s expected height based on age and sex. As an example, if we plot a person’s actual height on an age- and sex-specific growth chart, and this person’s height lies on the curve 1 standard deviation below the mean curve, that person has a height SDS of − 1. HD:SDS is the estimated difference between pubertal height SDS and adult height SDS [37]. A child who matures early is expected to be younger at PHV, resulting in a higher pubertal height SDS, finally leading to a higher HD:SDS than a child who matures at an older age, whereas a child who matures late has a lower HD:SDS [37].

Pubertal height SDS was obtained from height measures in the Children’s Database, a database initiated in 2009 aimed to systematically collect health related data on Danish children from school nurses and general practitioners [39]. Data for this study was extracted July 2017, and information on height was available on 51,751 of the 56,641 eligible children (91%). Pubertal height should be measured around the mean age of PHV [37]. Hence, we chose to include the height measure at an age nearest to 13 years in boys and 11 years in girls (+/− 2 years) based on Danish growth curves from 2014 [40]. Children with no information on height within these sex-specific time ranges were excluded (*n* = 7858, 14%). The height measures were converted to pubertal height SDS by use of the Danish growth curves [40], while excluding children with unreliable pubertal height SDS > 4 and < − 4 (*n* = 22). We were able to derive pubertal height SDS in 43,871 children (77%).

As the children had not reached their final adult height yet, adult height SDS was estimated by the following two steps. First, we estimated adult height based on a prediction algorithm, developed in a large Swedish study [41] using information on maternal and paternal height from the DNBC’s first interview during pregnancy. We excluded children with no information on maternal or paternal height (*n* = 1022). Second, we converted predicted adult height into adult height SDS using the Danish growth curves [40]. In total, we calculated HD:SDS for 23,003 boys and 19,846 girls with information on both pubertal and adult height SDS to obtain a final study sample of 42,849 children (76%) (Fig. 1).

### Information on other pubertal milestones

To estimate the validity of the modified HD:SDS, we used data on other pubertal milestones obtained from the Puberty Cohort, a nested cohort of 22,439 of the 56,641 eligible children in the present study [23]. To ensure statistical power, the Puberty Cohort was sampled from 12 different prenatal exposures of interest, including the three investigated in the present study, and combined with a random sample (*n* = 8000) of all eligible children. Based on rules from probability theory, we calculated each child’s probability of being sampled from the sampling fractions for each of the exposures and the random sample. The inverse of these probabilities were used as sampling weights to reweight the selected Puberty Cohort to represent a random sample of all 56,641 eligible children [23]. The Puberty Cohort was initiated in August 2012, and since then, 15,819 children (participation rate 70%) have participated (Fig. 1). Participants provided information on their pubertal development at least once either as part of the larger 11-year follow-up in the DNBC or as part of the shorter half-yearly, puberty-specific questionnaires from the age of 11.5 years. Data for the present study were extracted in January 2018. In the questionnaires, sons self-reported information on their current Tanner stage for pubic hair and genital development (stage 1–5), first ejaculation (yes/no; if yes: year and month), voice break (no/yes—sometimes/yes—definitive changes), acne (yes/no), and axillary hair (yes/no). Daughters self-reported information on Tanner stages for pubic hair and breast development (stage 1–5), menarche (yes/no; if yes: year and month), acne (yes/no), and axillary hair (yes/no). Information on Tanner stages was collected using the Sexual Maturation Scale, which includes line drawings of the five Tanner stages assisted by explanatory text [42–44].

### Prenatal exposures and covariates

We considered maternal pre-pregnancy body mass index (BMI), maternal smoking in pregnancy, and maternal alcohol intake in pregnancy as prenatal exposures. From the first telephone interview in the DNBC, we extracted information on all three prenatal exposures, which were self-reported by the mothers around gestational week 17. The covariate ‘highest social class of parents’ was classified according to the International Standard Class of Occupation and Education codes (ISCO-88 and ISCED) and obtained from Statistics Denmark. The covariates parity and maternal age at delivery were obtained from the Danish Medical Birth Registry, and the covariate maternal age at menarche was obtained from the first interview in the DNBC. Exposures and covariates were categorized as shown in Table 1. Childhood BMI was derived from height and weight reported by the mothers during the 7-year follow-up in the DNBC and was used in sub-analyses.

**Table 1:** Background characteristics of mothers to children born 2000–2003, the Danish National Birth Cohort, Denmark

Background Characteristics	Boys (*n* = 23,003) No. (%)	Girls (*n* = 19,846) No. (%)	Missing No. (%)
Pre-pregnancy BMI			614 (1.4)
< 18.5 kg/m^2^	984 (4.3)	833 (4.3)	
18.5–24.9 kg/m^2^	15,287 (67.4)	13,119 (67.1)	
25.0–29.9 kg/m^2^	4437 (19.6)	3875 (19.8)	
30.0+ kg/m^2^	1979 (8.7)	1721 (8.8)	
Smoking in pregnancy			41 (0.1)
Non-smoker	17,382 (75.6)	14,963 (75.5)	
Stopped smoking	2194 (9.5)	1933 (9.7)	
1–9 daily cigarettes	1921 (8.4)	1667 (8.4)	
10–14 daily cigarettes	949 (4.1)	768 (3.9)	
15+ daily cigarettes	532 (2.3)	499 (2.5)	
Alcohol in pregnancy			16 (0.0)
Abstainer	12,480 (54.3)	11,036 (55.6)	
< 1 weekly units	7199 (31.3)	6026 (30.4)	
1–3 weekly units	2787 (12.1)	2362 (11.9)	
> 3 weekly units	527 (2.3)	416 (2.1)	
Maternal age of menarche			373 (0.9)
Earlier than peers	5638 (24.7)	5036 (25.6)	
Same time as peers	13,359 (58.6)	11,278 (57.4)	
Later than peers	3819 (16.7)	3346 (17.0)	
Highest social class of parents			89 (0.2)
High grade professional	5565 (24.2)	4718 (23.8)	
Low grade professional	7271 (31.7)	6440 (32.5)	
Skilled worker	6499 (28.3)	5552 (28.0)	
Unskilled worker	3060 (13.3)	2620 (13.2)	
Student	442 (1.9)	379 (1.9)	
Economically inactive	115 (0.5)	99 (0.5)	
Parity			1 (0.0)
First child	11,146 (48.5)	9751 (49.1)	
Second child or more	11,856 (51.5)	10,095 (50.9)	
Maternal age at delivery, years, mean (sd)	30.5 (4.2)	30.4 (4.2)	23 (0.1)
Childhood BMI at 7 years, mean (sd)	15.7 (1.7)	15.6 (1.8)	17,008 (39.7)

*BMI* Body Mass Index, *sd* standard deviation

### Statistical analysis

To estimate the validity of our modified HD:SDS as a marker of pubertal timing, we estimated the correlations between HD:SDS and age at attaining the pubertal milestones collected in the Puberty Cohort (*n* = 12,061) (Fig. 1). For comparison, we also estimated the correlations between age at first ejaculation and age at attaining the other pubertal milestones in boys and between age at menarche and age at attaining the other pubertal milestones in girls. We chose age at first ejaculation and menarche as we had more precise information on these variables. As the information on the pubertal milestones were collected half-yearly, these data were censored (either right, left, or interval censored), except for age at first ejaculation and age at menarche that were uncensored for some children because specific ages were provided by the children. To account for the censoring, we used a time-to-event approach to obtain the correlations by maximum likelihood estimation under the assumption of a bivariate normal distribution using STATA’s -ml- package [45]. As the Puberty Cohort was sampled according to prenatal exposures of interest, we applied sampling weights, which have been described in detail previously [23].

In the main analysis, we used multivariable linear regression models for the three prenatal exposures to estimate difference in HD:SDS by exposure categories. To look for trends, we entered pre-pregnancy BMI (kg/m^2^), maternal smoking (categories as shown in Table 1), and alcohol consumption (units per week) as linear terms. Included potential confounders were highest social class of parents, parity, maternal age at delivery, maternal age at menarche, and the other exposures. Maternal age at delivery was included as a second order polynomial variable, while all other potential confounders was categorized as in Table 1 and included as indicator variables.

We conducted the following three sub-analyses. First, we assessed whether childhood BMI (continuous) modified the associations from the main analysis by including childhood BMI with interaction terms in each of the regression models as Houghton et al. found that postnatal growth patterns modified the association between maternal smoking and pubertal timing [46]. Second, we restricted the main analysis to having information on childhood BMI to assess possible bias due to missing information on childhood BMI (40%). Then, we further adjusted the main analysis for childhood BMI to assess potential mediation by childhood BMI. Third, we expanded the sex-specific age range for eligible height data from +/− 2 years to +/− 4 years around the mean age at PHV to reduce the number of excluded children without height data within the age range from 7858 (14%) to 1083 (2%).

Robust standard errors were used to account for clustering of siblings (*n* = 1116) and the use of sampling weights. STATA 15.1 MP software (Statacorp, College Station, TX) was used for all analyses.

## Results

### Study population

Mean age (standard deviation) at height measures during puberty was 12.88 (0.98) years in boys and 11.50 (0.89) years in girls. Characteristics of the mother-child pairs are presented in Table 1.

When comparing mothers of children with information on HD:SDS to mothers of children without information on HD:SDS, maternal pre-pregnancy BMI, alcohol intake, maternal age at menarche, parity, and maternal age at delivery were similar (prevalence difference < 2% on any of these variables). Mothers of children with information on HD:SDS were slightly more likely to be non-smokers (75.6% vs 71.9%) and to work as a high grade professional (24.0% vs 21.9%) than mothers of children without information on HD:SDS. For the Puberty Cohort subsample, the only difference was a slightly higher proportion of mothers of children with information on HD:SDS were non-smokers (75.4% vs 72.0%), whereas all other characteristics were equally distributed across children with and without information on HD:SDS.

### Validation of HD:SDS

The correlation coefficients between HD:SDS and age at attaining the pubertal milestones ranged from − 0.20 to − 0.53 for boys and from − 0.30 to − 0.53 for girls (Table 2). The correlations between age at first ejaculation and age at attaining the other pubertal milestones in boys ranged from 0.28 to 0.41, and the correlations between age at menarche and age at attaining the other pubertal milestones in girls ranged from 0.37 to 0.71.

**Table 2:** Correlations for HD:SDS, first ejaculation, and menarche with age at attaining other pubertal milestones in boys and girls born 2000–2003, the Puberty Cohort, Denmark

	Correlations for HD:SDS			Correlations for first ejaculation (boys) and menarche (girls)		
Pubertal milestones	No.^a^	Estimate	95% CI	No.	Estimate	95% CI
Boys						
Tanner Genital Stage 2	6175	−0.26	−0.29, − 0.22	7679	0.28	0.24, 0.31
Tanner Genital Stage 3	6175	− 0.39	− 0.41, − 0.36	7679	0.36	0.32, 0.39
Tanner Genital Stage 4	6175	−0.44	− 0.46, − 0.41	7679	0.38	0.35, 0.41
Tanner Genital Stage 5	6175	−0.32	− 0.35, − 0.28	7679	0.28	0.24, 0.31
Tanner Pubic Hair Stage 2	6179	−0.30	− 0.33, − 0.27	7679	0.30	0.26, 0.33
Tanner Pubic Hair Stage 3	6179	−0.45	− 0.47, − 0.42	7679	0.37	0.34, 0.40
Tanner Pubic Hair Stage 4	6179	−0.53	− 0.56, − 0.51	7679	0.41	0.38, 0.44
Tanner Pubic Hair Stage 5	6179	−0.44	− 0.47, − 0.41	7679	0.37	0.34, 0.41
Axillary Hair	6185	−0.39	− 0.42, − 0.36	7679	0.28	0.24, 0.31
Acne	6185	−0.31	− 0.34, − 0.28	7679	0.28	0.24, 0.31
Voice Break	6015	−0.34	− 0.37, − 0.31	7485	0.34	0.31, 0.37
First Ejaculation	6171	−0.20	− 0.24, − 0.17		Ref	
Girls						
Tanner Breast Stage 2	5866	−0.50	−0.54, − 0.47	8111	0.63	0.61, 0.66
Tanner Breast Stage 3	5866	−0.53	− 0.55, − 0.50	8111	0.71	0.69, 0.72
Tanner Breast Stage 4	5866	−0.45	−0.48, − 0.42	8111	0.65	0.63, 0.67
Tanner Breast Stage 5	5866	−0.33	− 0.36, − 0.29	8111	0.46	0.43, 0.48
Tanner Pubic Hair Stage 2	5867	−0.49	−0.52, − 0.46	8111	0.61	0.59, 0.64
Tanner Pubic Hair Stage 3	5867	−0.48	− 0.50, − 0.45	8111	0.64	0.62, 0.66
Tanner Pubic Hair Stage 4	5867	−0.37	− 0.40, − 0.34	8111	0.52	0.50, 0.55
Tanner Pubic Hair Stage 5	5867	−0.30	− 0.34, − 0.27	8111	0.40	0.37, 0.43
Axillary Hair	5870	−0.40	− 0.43, − 0.37	8111	0.48	0.45, 0.50
Acne	5870	−0.30	− 0.33, − 0.27	8111	0.37	0.34, 0.40
Menarche	5864	−0.53	− 0.55, − 0.51		Ref	

*Abbreviations*: *CI* Confidence interval, *HD:SDS* Height difference in standard deviations, *Ref* Reference

^a^As some boys and girls gave information on some but not all pubertal milestones, different number of observations were used for each outcome

### Prenatal exposures

With normal weight (BMI: 18.5–24.9 kg/m^2^) as the reference, maternal pre-pregnancy obesity (BMI: 30.0+ kg/m^2^) was associated with higher HD:SDS (boys: 0.23 (95% confidence interval (CI): 0.18, 0.28); girls: 0.19 (95% CI: 0.14, 0.24)) indicating earlier pubertal timing in both sexes (Table 3). Maternal pre-pregnancy overweight (BMI: 25.0–29.9 kg/m^2^) was also associated with higher HD:SDS in both boys and girls. Dose-dependent associations were observed across BMI groups for both boys and girls. Maternal smoking in pregnancy was not associated with HD:SDS. Compared to maternal alcohol abstainers, intake of more than 3 weekly units of alcohol during pregnancy was associated with lower HD:SDS in boys only (− 0.14 (95% CI, − 0.24, − 0.05)) indicating delayed pubertal timing.

**Table 3:** Prenatal exposures and pubertal timing, measured by HD:SDS^a^, in children born 2000–2003, the Danish National Birth Cohort, Denmark

	Boys (*n* = 22,409)			Girls (*n* = 19,301)		
	Crude	Adjusted^b^		Crude	Adjusted^b^	
Prenatal exposures	Difference	Difference	95% CI	Difference	Difference	95% CI
Pre-pregnancy BMI						
< 18.5 kg/m^2^	−0.15	− 0.13	− 0.19, − 0.06	− 0.10	− 0.07	− 0.14, − 0.01
18.5–24.9 kg/m^2^	Ref	Ref		Ref	Ref	
25.0–29.9 kg/m^2^	0.17	0.12	0.09, 0.16	0.15	0.10	0.06, 0.13
30.0+ kg/m^2^	0.30	0.23	0.18, 0.28	0.26	0.19	0.14, 0.24
Trend analysis (kg/m^2^)	0.027	0.021	0.018, 0.024	0.025	0.019	0.015, 0.022
Smoking in pregnancy						
Non-smoker	Ref	Ref		Ref	Ref	
Stopped smoking	−0.01	−0.01	−0.05, 0.04	− 0.03	−0.04	− 0.08, 0.01
1–9 daily cigarettes	−0.01	− 0.02	− 0.07, 0.03	0.05	0.03	−0.02, 0.08
10–14 daily cigarettes	0.05	0.01	−0.06, 0.08	0.09	0.04	−0.03, 0.11
15+ daily cigarettes	0.03	−0.02	−0.11, 0.07	0.11	0.02	−0.07, 0.11
Trend analysis (group)	0.007	−0.003	−0.017, 0.011	0.023	0.008	−0.007, 0.022
Alcohol in pregnancy						
Abstainers	Ref	Ref		Ref	Ref	
< 1 units weekly	− 0.01	0.00	−0.03, 0.03	−0.03	−0.02	−0.05, 0.01
1–3 units weekly	− 0.05	− 0.02	− 0.07, 0.02	−0.05	− 0.02	−0.07, 0.02
> 3 units weekly	− 0.16	− 0.14	− 0.24, − 0.05	− 0.01	0.00	−0.10, 0.09
Trend analysis (units)	−0.027	−0.021	−0.035, − 0.008	− 0.013	− 0.006	− 0.019, 0.007

*Abbreviations*: *BMI* Body Mass Index, *CI* Confidence interval, *Ref* Reference, *HD:SDS* Height difference in standard deviations

^a^A negative HD:SDS indicates later pubertal timing, and a positive HD:SDS indicates earlier pubertal timing

^b^Adjusted for highest social class of parents, parity, maternal age at delivery, maternal age at menarche and all other exposures (in categories) in this table

In sub-analyses, we found no statistically significant interaction between childhood BMI and maternal lifestyle exposures (*P*-values ranged from 0.11 and 0.98). When restricting to having information on childhood BMI, the results remained essentially unchanged (Model 1, Table 4). When further adjusting for childhood BMI, the associations for pre-pregnancy BMI were attenuated, the associations for maternal smoking in pregnancy shifted towards lower HD:SDS, and the associations for maternal alcohol intake in pregnancy remained unchanged (Model 2, Table 4). When expanding the sex-specific age range for eligible height data from +/− 2 years to +/− 4 years, the results for the main analysis remained essentially unchanged (Additional file 1: Table S1).

**Table 4:** Prenatal exposures and pubertal timing, measured by HD:SDS^a^, when further adjusting for childhood BMI at 7 years in children born 2000–2003, the Danish National Birth Cohort, Denmark

	Model 1^b^: Adjusted difference in HD:SDS when restricting to information on childhood BMI		Model 2^c^: Adjusted difference in HD:SDS when also adjusting for childhood BMI	
Prenatal exposures	Difference	95% CI	Difference	95% CI
Boys (*n* = 13,674)				
Pre-pregnancy BMI				
< 18.5 kg/m^2^	−0.17	−0.26, − 0.08	− 0.12	− 0.20, − 0.03
18.5–24.9 kg/m^2^	Ref		Ref	
25.0–29.9 kg/m^2^	0.12	0.07, 0.16	0.06	0.01, 0.10
30.0+ kg/m^2^	0.24	0.18, 0.31	0.13	0.06, 0.19
Trend analysis (kg/m^2^)	0.023	0.018, 0.027	0.012	0.007, 0.016
Smoking in pregnancy				
Non-smoker	Ref		Ref	
Stopped smoking	0.00	−0.05, 0.06	−0.02	−0.07, 0.04
1–9 daily cigarettes	−0.01	−0.07, 0.06	−0.05	−0.12, 0.01
10–14 daily cigarettes	0.01	−0.09, 0.11	−0.04	−0.13, 0.06
15+ daily cigarettes	0.01	−0.13, 0.14	−0.05	−0.18, 0.08
Trend analysis (group)	0.001	−0.018, 0.021	−0.016	−0.036, 0.003
Alcohol in pregnancy				
Abstainers	Ref		Ref	
< 1 units weekly	0.00	−0.04, 0.04	0.00	−0.04, 0.04
1–3 units weekly	0.00	−0.06, 0.05	0.00	−0.05, 0.05
> 3 units weekly	−0.12	−0.23, − 0.01	− 0.11	− 0.22, 0.00
Trend analysis (units)	−0.018	−0.035, − 0.002	− 0.017	− 0.033, 0.000
Girls (*n* = 11,550)				
Pre-pregnancy BMI				
< 18.5 kg/m^2^	−0.02	−0.11, 0.07	0.05	−0.04, 0.14
18.5–24.9 kg/m^2^	Ref		Ref	
25.0–29.9 kg/m^2^	0.10	0.06, 0.15	0.04	−0.01, 0.08
30.0+ kg/m^2^	0.16	0.09, 0.24	0.05	−0.02, 0.11
Trend analysis (kg/m^2^)	0.018	0.014, 0.023	0.007	0.002, 0.011
Smoking in pregnancy				
Non-smoker	Ref		Ref	
Stopped smoking	−0.05	−0.11, 0.00	−0.08	−0.14, − 0.02
1–9 daily cigarettes	0.03	−0.04, 0.10	−0.03	−0.09, 0.04
10–14 daily cigarettes	0.05	−0.06, 0.15	0.00	−0.10, 0.10
15+ daily cigarettes	0.04	−0.10, 0.19	−0.02	−0.16, 0.12
Trend analysis (group)	0.009	−0.012, 0.029	−0.012	−0.032, 0.008
Alcohol in pregnancy				
Abstainers			Ref	
< 1 units weekly	−0.03	−0.07, 0.01	−0.03	−0.07, 0.01
1–3 units weekly	−0.06	−0.11, 0.00	−0.06	−0.12, − 0.01
> 3 units weekly	0.02	−0.11, 0.14	0.00	−0.12, 0.12
Trend analysis (units)	−0.012	−0.029, 0.006	−0.014	−0.031, 0.003

*Abbreviations*: *BMI* Body Mass Index, *CI* Confidence interval, *Ref* Reference, *HD:SDS* Height difference in standard deviations

^a^A negative HD:SDS indicates later pubertal timing, and a positive HD:SDS indicates earlier pubertal timing

^b^Model 1 is restricted to having information on childhood BMI and adjusted for highest social class of parents, parity, maternal age at delivery, maternal age at menarche and all other exposures (in categories) in this table

^c^Model 2 is adjusted for childhood BMI, highest social class of parents, parity, maternal age at delivery, maternal age at menarche and all other exposures (in categories) in this table

## Discussions

HD:SDS was moderately correlated with age at attaining other pubertal milestones. When using HD:SDS as a marker of pubertal timing, higher maternal pre-pregnancy BMI was associated with earlier pubertal timing in both boys and girls. Maternal alcohol consumption during pregnancy was associated with later pubertal timing in boys only, whereas maternal smoking was not associated with pubertal timing using HD:SDS. We found no evidence of interaction between childhood BMI and the three prenatal exposures. Childhood BMI seemed to mediate the association between pre-pregnancy BMI and HD:SDS.

### Interpretation

Both HD:SDS and age at PHV capture the timing of accelerated linear growth in puberty. This is supported by strong correlations between HD:SDS and age at PHV of 0.84 in boys and 0.78 in girls [37]. However, age at PHV has large variability across Tanner stages [47], and lower correlations for both HD:SDS and PHV with the other pubertal milestones are expected. This is supported by a recent study that reported correlations of 0.27 between age at PHV and onset of breast development and 0.48 between age at PHV and menarche [48]. This is comparable to the correlations around − 0.30 to − 0.50 for HD:SDS and age at attaining the majority of the pubertal milestones in the present study. In comparison, the correlations between age at first ejaculation and the other pubertal milestones in boys were in the same range, although the correlations between age at menarche and the other pubertal milestones in girls were slightly higher. As the correlations between HD:SDS and age at attaining the pubertal milestones were within the expected ranged, the validity of our modified version of HD:SDS as a marker of pubertal timing seems acceptable in large epidemiological studies.

We found that exposure to maternal pre-pregnancy obesity and overweight was associated with higher HD:SDS indicating earlier pubertal timing in both boys and girls. These associations have been well-documented in girls [13–19]. In comparison, the only study so far in boys reported earlier age at regular shavings but no difference in age at voice break, first nocturnal ejaculation, and acne in sons of obese mothers [31]. That study was, however, limited by self-reported information on puberty when the boys were 18 to 21 years old [31]. Obese mothers are at increased risk of having obese children [9, 49], which, in turn, is associated with earlier puberty [4]. Hence, childhood obesity may play a mediatory role for the observed associations. When we adjusted for childhood BMI in the present study, the results attenuated supporting a mediatory role of child BMI in line with some [15, 16, 19] but not all studies [13, 14] in girls.

In a recent study from the Puberty Cohort, maternal smoking during pregnancy was consistently associated with earlier age at reaching a wide range of pubertal markers in both boys and girls [23]. Maternal smoking was, however, not associated with pubertal timing when measured by HD:SDS. We speculate that this null finding may be induced by the following biasing mechanism. Prenatal smoking has been associated with impaired growth throughout life [50] and may, therefore, result in lower pubertal height SDS and lower adult height SDS. When predicting adult height SDS from parental height, we, therefore, could be overestimating adult height SDS in children of smoking mothers because the prediction algorithm does not account for the shorter final adult height due maternal smoking in pregnancy. This might lead to a downward bias of HD:SDS among children of smoking mothers that could potentially cancel out the expected association with maternal smoking [23]. In a sub-analysis, we also adjusted for childhood BMI, and the associations for maternal smoking shifted towards lower HD:SDS, although with more uncertainty. This finding warrants a cautious interpretation due to the possible bias described above.

For boys prenatally exposed to maternal alcohol, we observed a smaller HD:SDS, indicating later pubertal timing. Moreover, the association remained unchanged after adjustment for childhood BMI, indicating that other pathways may be at play. So far, studies on maternal alcohol intake during pregnancy have found no association with pubertal timing in boys when using average weekly intake as the exposure [26, 32, 33]. One of these studies, however, reported a tendency towards later pubertal timing when prenatally exposed to ≥5 episodes of binge drinking [33]. A potential delaying effect on pubertal timing by prenatal alcohol is supported by animal studies that have reported smaller testes, lower postnatal androgens, shorter anogenital distance, and delayed markers of pubertal timing in male rats prenatally exposed to alcohol [51–53]. Likewise, prenatal alcohol seems to delay pubertal timing in female mice and rats [53–55]. However, most epidemiologic studies on pubertal timing in girls after moderate prenatal alcohol exposure have found no association, which is in line with our results [22, 24–26]. The reason for this discrepancy between sexes might be that boys are more prone to morbidity as neonates than girls. Although speculative, this might explain that we only observe an association for boys at the exposure level in our study. In line with this, girls exposed to high maternal alcohol intake (≥14 units per week) were found to experience later menarche in one study, although that study was small with no adjustment for confounders [27]. Thus, we cannot exclude a delaying effect of prenatal exposure to higher levels of alcohol in girls.

Over the last decades, trends towards reduced alcohol intake during pregnancy [35, 56, 57] and increased prevalence of obesity have been observed [36]. Whereas the association between prenatal exposure to alcohol and pubertal timing in boys needs to be replicated, increased prevalence of obesity during pregnancy might explain some of the trend in pubertal timing.

### Strengths and limitations

The main strengths of the present study were its large size, the use of pubertal height data measured by health care professionals, and the validation of HD:SDS using information on other pubertal milestone from a puberty cohort. An important limitation was that the children were not fully grown, and we, therefore, had to rely on predictions based on parental height, which lead to some measurement error. This is especially important if the exposures affect postnatal growth. If so, the prediction algorithm for adult height may systematically overestimate or underestimate adult height among the exposed leading to biased estimates. This is possibly the case for maternal smoking as discussed above. As obese children generally achieve normal adult height [4], we speculate that children exposed in utero to maternal pre-pregnancy obesity will most likely also achieve normal adult height. Similarly, the relatively low exposure level to prenatal alcohol in the present study will probably not interfere with growth in the children, although heavy prenatal alcohol exposure may lead to growth restriction, a feature of Fetal Alcohol Syndrome [8]. Hence, the estimates for maternal pre-pregnancy BMI and alcohol intake need not be systematically biased. Under more restricted conditions such as poverty or famine, HD:SDS might break down as a marker of pubertal timing because the children might not reach their full genetic growth potential during childhood, puberty, and adulthood. Due to the observational nature of this study, we cannot rule out that residual confounding or confounding from unmeasured factors, such as diet or genetic factors, have influenced the results. Even though we had information on HD:SDS for 76% of the participants, we cannot rule out the risk of selection bias. However, the background characteristics were relatively evenly distributed across children with and without information on HD:SDS. Including further 12% more children in the main analysis (participation rate 89%), by expanding the sex-specific age range for having eligible height data, did not change the results. The relatively late start of follow-up at 11 years resulted in a relatively large proportion of left-censored observations for the earliest milestones, which may bias the correlations between HD:SDS and the age at attaining the earliest pubertal milestones if the assumption of the bivariate normal distribution is violated. For the earliest milestones, such as Tanner Breast stage 2, it is not possible to assess the assumption of normality as we do not have information on the left 85% of the distribution. However, all later milestones were compatible with the normal distribution [58].

## Conclusions

HD:SDS appears to be a useful marker of pubertal timing in large epidemiological studies. Maternal pre-pregnancy obesity and overweight were associated with earlier timing of puberty in both boys and girls, while maternal alcohol intake in pregnancy was associated with delayed puberty in boys. When relying on predicted adult height SDS from parental data, a cautious interpretation is needed for prenatal exposures that interfere with postnatal growth. This may explain why the present study did not replicate the previously reported association between maternal smoking and early pubertal timing.

## Supplementary information

**Additional file 1: Table S1.** Sub-analysis: Prenatal exposures and pubertal timing, measured by HD:SDS^a^, when including children with height data +/− 4 years around the man age at peak height velocity in children born 2000–2003, the Danish National Birth Cohort, Denmark.

## Data Availability

The dataset analyzed in the study is not publicly available due to national data security legislation on sensitive personal data. Access to data can be applied for at dnbc-research@ssi.dk.

## Abbreviations

BMI: Body mass index
CI: Confidence interval
DNBC: Danish National Birth Cohort
HD:SDS: Height difference in standard deviations
PHV: Peak height velocity
SDS: Standard deviation scores
