# Supplementary material for: Maternal pre-pregnancy body mass index, smoking in pregnancy, and alcohol intake in pregnancy in relation to pubertal timing in the children
Source: BMC Pediatr. 2019 Sep 16;19:338. doi: 10.1186/s12887-019-1715-0 (PMC6745800; doi:10.1186/s12887-019-1715-0)
Supplement: Supplementary file 1 — Additional file 1: Table S1. Sub-analysis: Prenatal exposures and pubertal timing, measured by HD:SDSa, when including children with height data +/− 4 years around the man age at peak height velocity in children born 2000–2003, the Danish National Birth Cohort, Denmark. [file 12887_2019_1715_MOESM1_ESM.docx]

| Additional file 1: Table S1. Sub-analysis: Prenatal exposures and pubertal timing, measured by HD:SDS^a^, when including children with height data +/− 4 years around the man age at peak height velocity in children born 2000-2003, the Danish National Birth Cohort, Denmark. | | | | | | | |
| --- | --- | --- | --- | --- | --- | --- | --- |
|  | Boys (n = 24 884) | | |  | Girls (n = 23 258) | | |
|  | Crude | Adjusted^b^ | |  | Crude | Adjusted^b^ | |
| Prenatal exposures | Difference | Difference | 95% CI |  | Difference | Difference | 95% CI |
| Pre-pregnancy BMI |  |  |  |  |  |  |  |
| <18.5 kg/m^2^ | −0.16 | -0.13 | -0.19, -0.07 |  | -0.11 | -0.09 | -0.15, -0.03 |
| 18.5-24.9 kg/m^2^ | Ref | Ref |  |  | Ref | Ref |  |
| 25.0-29.9 kg/m^2^ | 0.16 | 0.12 | 0.08, 0.15 |  | 0.14 | 0.10 | 0.07, 0.13 |
| 30.0+ kg/m^2^ | 0.28 | 0.21 | 0.16, 0.26 |  | 0.24 | 0.17 | 0.13, 0.22 |
| Trend analysis (kg/m^2^) | 0.026 | 0.020 | 0.017, 0.023 |  | 0.024 | 0.018 | 0.015, 0.021 |
| Smoking in pregnancy |  |  |  |  |  |  |  |
| Non-smoker | Ref | Ref |  |  | Ref | Ref |  |
| Stopped smoking | -0.01 | -0.01 | -0.05, 0.04 |  | -0.02 | -0.03 | -0.07, 0.01 |
| 1-9 daily cigarettes | 0.00 | -0.01 | -0.06, 0.03 |  | 0.03 | 0.01 | -0.04, 0.05 |
| 10-14 daily cigarettes | 0.05 | 0.01 | -0.05, 0.08 |  | 0.07 | 0.02 | -0.04, 0.09 |
| 15+ daily cigarettes | 0.02 | -0.03 | -0.11, 0.06 |  | 0.09 | 0.01 | -0.07, 0.10 |
| Trend analysis (group) | 0.007 | -0.003 | -0.016, 0.011 |  | 0.018 | 0.003 | -0.010, 0.016 |
| Alcohol in pregnancy |  |  |  |  |  |  |  |
| Abstainers | Ref | Ref |  |  | Ref | Ref |  |
| <1 units weekly | -0.01 | 0.01 | -0.02, 0.03 |  | -0.02 | -0.01 | -0.04, 0.02 |
| 1-3 units weekly | -0.05 | -0.03 | -0.07, 0.01 |  | -0.05 | -0.02 | -0.06, 0.02 |
| >3 units weekly | -0.14 | -0.13 | -0.22, -0.04 |  | -0.01 | -0.01 | -0.09, 0.08 |
| Trend analysis (units) | -0.026 | -0.021 | -0.034, -0.008 |  | -0.010 | -0.005 | -0.017, 0.007 |
| Abbreviations: BMI, Body Mass Index; CI, confidence interval; Ref, reference; HD:SDS, height difference in standard deviations.  ^a^A negative HD:SDS indicates later pubertal timing, and a positive HD:SDS indicates earlier pubertal timing.  ^b^Adjusted for highest social class of parents, parity, maternal age at delivery, maternal age at menarche and all other exposures (in categories) in this table. | | | | | | | |
